# Side Effects of COVID-19 Inactivated Virus vs. Adenoviral Vector Vaccines: Experience of Algerian Healthcare Workers

**DOI:** 10.3389/fpubh.2022.896343

**Published:** 2022-05-16

**Authors:** Mohamed Lounis, Mohammed Amir Rais, Djihad Bencherit, Hani Amir Aouissi, Adda Oudjedi, Jitka Klugarová, Andrea Pokorná, Miloslav Klugar, Abanoub Riad

**Affiliations:** ^1^Department of Agro-Veterinary Science, Faculty of Natural and Life Sciences, University of Ziane Achour, Djelfa, Algeria; ^2^Department of Dentistry, Faculty of Medicine, University of Algiers Benyoucef Benkhedda, Algiers, Algeria; ^3^Department of Biology, Faculty of Natural and Life Sciences, University of Ziane Achour, Djelfa, Algeria; ^4^Scientific and Technical Research Center on Arid Regions (CRSTRA), Biskra, Algeria; ^5^Institute of Science and Techniques of Physical and Sport Activities, Larbi Ben M'hidi University, Oum El Bouaghi, Algeria; ^6^Czech National Center for Evidence-Based Healthcare and Knowledge Translation (Cochrane Czech Republic, Czech EBHC: JBI Center of Excellence, Masaryk University GRADE Center), Faculty of Medicine, Institute of Biostatistics and Analyses, Masaryk University, Brno, Czechia; ^7^Department of Health Sciences, Faculty of Medicine, Masaryk University, Brno, Czechia; ^8^Department of Public Health, Faculty of Medicine, Masaryk University, Brno, Czechia

**Keywords:** adenoviral-based vaccine, COVID-19, health workers, inactivated virus vaccine, side effects

## Abstract

Healthcare workers were prioritized in vaccination campaigns globally because they are exposed to the highest risk of contamination by SARS-CoV-2. This study evaluated the self-reported post-vaccination side effects of inactivated (BBIBP-CorV and CoronaVac) and adenoviral vector-based (AZD1222, Gam-COVID-Vac and Ad26.COV2.S) vaccines among Algerian healthcare workers using a validated questionnaire. The final analysis included 721 healthcare workers, with a predominance of females (59.1%) and younger individuals 20–30 years old (39.4%). Less than half (49.1%) of the respondents reported at least one local side effect, while 53.8% reported at least one systemic side effect. These side effects were more prevalent among viral vector vaccinees than inactivated virus vaccinees. The most common local side effects were injection site pain (39%) and arm pain (25.4%), while fatigue (34.4%), fever (28.4%), headache (24.8%) and myalgia (22.7%) were the most prevalent systemic side effects. The side effects appeared earlier among inactivated virus vaccines recipients and generally lasted for 2 to 3 days for the two vaccinated groups. The risk factors associated with a higher prevalence of side effects included female gender, allergic individuals, individuals with regular medication, those who contracted the COVID-19 disease and those who received two doses for both inactivated and viral-based vaccines groups. Despite the higher prevalence of post-vaccination side effects among adenoviral vector vaccines recipients, both vaccines groups were equally effective in preventing symptomatic infections, and no life-threatening side effects were reported in either vaccine group.

## Introduction

As of March 2022, four hundred and forty-one million cases and nearly six million fatalities were recorded globally due to the coronavirus disease (COVID-19) pandemic (1). After the second anniversary of its emergence, the disease continues its rapid spread despite the drastic preventive measures applied in all countries worldwide. In the absence of vaccines or efficient medications against this disease during the first wave, countries had no alternatives other than non-pharmacological preventive measures like lockdowns, travel restrictions, physical distancing, quarantine, and using face masks to limit the disease propagation according to their capacities (2). These measures have helped to limit the propagation of the disease; however, they seem to be insufficient to control the disease entirely, and the COVID-19 resurged in multiple waves when countries started their deconfinement (3, 4).

Hence, researchers were racing against the clock to find the best strategy to fight this disease and return to normal life. In this way, herd immunity or population immunity through vaccination or immunity developed after a previous infection was one of the proposed strategies (5). Given the impossibility to achieve herd immunity through natural infection, the best approach to achieve herd immunity recommended by the World Health Organization (WHO) is to protect people by vaccination (6, 7). These exceptional circumstances have pushed researchers and laboratories to develop and produce different types of vaccines in a short period of about 1 year (8). In December 2020, the World Health Organization (WHO) had approved six vaccines types, and the mass vaccination campaign started since then (9).

Currently, 35 COVID-19 vaccines are approved by at least one country, and ten vaccines are approved by the WHO (9). However, myths, speculations, misinformation and conspiracy theories surrounding COVID-19 vaccines and their side effects have highly influenced vaccine uptake. These factors have caused delays due to unwillingness in people to get vaccinated, leading to vaccine hesitancy (10–14). Multiples studies have reported that this hesitancy is mainly related to vaccines' safety and effectiveness; however, all approved vaccines had high efficacy levels (10–17). Nevertheless, like any other pharmacological agents, these vaccines could induce some side effects that could include flu-like symptoms (e.g. headache, fatigue and myalgia) and injection site reactions and are mostly non-serious and of short duration (18–28).

Algeria started its mass vaccination campaign on December 31, 2020. The vaccines had been administered first to healthcare workers and individuals with comorbidities (29–31). Currently, the approved vaccines in the country include inactivated virus vaccines, i.e., BBIBP-CorV and CoronaVac, and adenoviral vector-based vaccines, i.e., Gam-COVID-Vac, AZD1222 and Ad26.COV2.S (30–32). On February 20, 2022, more than 7.46 million persons received at least one dose of COVID-19, representing about 16.7% of the total population (33).

The current work was conducted to determine the most common side effects reported by healthcare workers in Algeria after COVID-19 vaccination and to evaluate eventual risk factors associated with post-vaccination side effects. To the best of our knowledge, no such studies about COVID-19 vaccine side effects were conducted in Algeria.

## Materials and Methods

### Design

The present study had been designed as an analytical cross-sectional survey-based study that utilized a self-administered questionnaire (SAQ) to collect data from the target population about their post-vaccination side effects. The study was designed and reported according to the STrengthening the Reporting of OBservational studies in Epidemiology (STROBE) guidelines for cross-sectional studies (34).

### Setting

This study was carried out between October 25 and November 25, 2021 after 6,328,806 (14.4%) of the Algerian population received at least one dose and 4,751,933 (10.8%) were fully vaccinated in order to ensure that a substantial proportion of the Algerian healthcare workers were already vaccinated. The study utilized a SAQ that was designed and administered digitally using Google Forms (Google LLC, Menlo Park, CA, USA, 2021)(35). A uniform resource locator (URL) and a quick response (QR) code were used to disseminate the SAQ and collect data from the target population.

### Participants

The target population of this study were Algerian healthcare workers who received either one or two doses of COVID-19 vaccines that were approved for mass inoculation in Algeria. The participants who received inactivated virus and adenoviral vector vaccines were included, while the participants who received protein sub-unit mRNA-based vaccines were excluded from the subsequent analyses.

A non-random technique through convenience sampling was used as the potential participants were recruited using social media platforms (Facebook and WhatsApp groups) targeting especially those of medical interests.

Epi-Info ^TM^ version 7.2.4 (CDC. Atlanta, GA, USA, 2020) had been used to calculate the sample size using the following assumptions of an expected outcome frequency of 50%, an acceptable margin error of 4%, a confidence level (CI) of 95%, and a postulated proportion of responses resulted from careless/insufficient effort (C/IE) of 10%(36). The required sample size for this study was 660 responses.

Participation in this study was on voluntary basis and it was not incentivised by financial rewards or any other means of compensation. The participants' identity was kept anonymous in order to control Hawthorne's effect and information bias.

### Instrument

The SAQ used in this study was adopted from previous studies and its items had been reviewed by a panel of experts to assess content validity. Consequently, test re-test reliability of the items was estimated to be acceptable with a mean Cohen's kappa coefficient of 0.89 ± 0.13 and reported in detail previously (23–28, 37). The SAQ comprised 25 multiple choice items that were stratified into three categories; (i) demographic characteristics including sex, age group, and profession, (ii) anamnestic characteristics including chronic illnesses, medications, allergies, previous COVID-19 infection, and COVID-19 vaccine type and number of doses, and (iii) post-vaccination side effects, their onset and duration, and post-vaccination medical care and medications.

### Ethics

The study protocol had been reviewed and approved by the Scientific Committee of the Faculty of Natural and Life Sciences/University of Djelfa on 20/10/2021 with the reference number 117/10/2021. The Declaration of Helsinki for research involving human subjects had guided the conception and execution of the entire study (38). All participants provided their informed consent digitally before filling the questionnaire. The responses of the participants who did not complete the questionnaire were not saved; and the participants were able to leave the study any time without justification. Given the fact that no identifying personal data was collected, retrospective identification of the participants was not possible.

### Analyses

The Statistical Package for the Social Sciences (SPSS) version 28.0 (SPSS Inc. Chicago, IL, USA, 2021) was used to analyse the collected data (39). Initially, descriptive statistics used frequencies (*n*) and percentages (%) to summarize nominal and ordinal data. Then, inferential statistics through chi-squared test (χ^2^) and Fisher's-exact test had been used to evaluate the association between independent and dependent variables. Eventually, multivariable logistic regression was used to evaluate the suggested risk factors of post-vaccination side effects following inactivated virus vaccines and adenoviral vector vaccines. All analytical tests were performed with a confidence level (*CI*) of 95% and a significance level (*Sig*.) of ≤ 0.05.

## Results

### Demographic Characteristics

A total of 724 responses were received during the study period (October 25–November 25, 2021), of which three responses were excluded because the respondents received mRNA-based vaccines (two received BNT162b2 and one received mRNA-1273).

Out of the 721 included participants, 450 received BBIBP-CorV or CoronaVac (inactivated virus group, *n* = 450), while 156 received Gam-COVID-Vac, 98 received AZD1222, and 17 received Ad26.COV2.S (adenoviral vector group, *n* = 271).

The most commonly represented age group was the 20–30 years-old (39.4%), followed by the 31–40 years-old (31.8%) and the 41–50 years-old (17.5%) Table 1.

**Table 1:** Demographic and anamnestic characteristics of Algerian healthcare workers receiving COVID-19 vaccines (*n* = 721).

**Variable**	**Outcome**	**Frequency** **(*n*)**	**Percentage** **(*%*)**
**Sex**	Female	426	59.1%
	Male	295	40.9%
**Age group**	20–30 years-old	284	39.4%
	31–40 years-old	229	31.8%
	41–50 years-old	126	17.5%
	51–60 years-old	66	9.2%
	> 60 years-old	16	2.2%
**Tobacco**	Smoker	86	11.9%
**smoking**	Non-smoker	635	88.1%
**Chronic**	Autoimmune disorders	5	0.7%
**illnesses**	Cardiovascular disease	9	1.2%
	Chronic hypertension	59	8.2%
	COPD	33	4.6%
	Diabetes mellitus	39	5.4%
	Gastrointestinal disease	3	0.4%
	Thyroid disorders	16	2.2%
	Others	43	6%
	Total	168	23.3%
**Allergy**	Yes	218	30.2%
	No	503	69.8%
**Medications**	Anti-asthma	38	5.3%
	Anticoagulants	4	0.6%
	Antidepressants	13	1.8%
	Anti-diabetes	35	4.9%
	Antihistamines	112	15.5%
	Antihypertensive	55	7.6%
	Anti-reflux	36	5%
	Cholesterol-lowering	10	1.4%
	Contraceptives	20	2.8%
	Thyroid hormone	34	4.7%
	Total	276	38.3%

More than half (54.4%) of the sample were married, while 45.1% were single and 0.4% were either divorced or widow. Physicians (35.5%) were the most participating profession, followed by dentists (20.4%), nurses (9.3%), paramedics (9.3%), and pharmacists (7.4%). Most participants worked for public (state-funded) healthcare providers (77.3%). The most contributing department was Algiers (25.2%), followed by Blida (5.7%), Tebessa (4.8%), Oran (4.7%), Sétif (4%), Annaba (3.9%), and Constantine (3.6%) Supplementary Table 1.

### Anamnestic Characteristics

A total of 11.9% of the participants reported smoking tobacco regularly with no significant (*Sig*. = 0.526) difference between inactivated virus (11.3%) and adenoviral vector (12.9%) groups. Chronic hypertension was the most commonly reported chronic illness (8.2%), followed by diabetes mellitus (5.4%), and chronic obstructive pulmonary disease (4.6%). Overall, 23.3% of the participants reported suffering from at least one chronic illness, and 30.2% reported having allergy to at least one allergen with no significant differences between inactivated virus and adenoviral vector groups.

The most commonly administered medications were antihistamines (15.5%), followed by antihypertensive drugs (7.6%), anti-asthma (5.3%), anti-reflux (5%), anti-diabetes drugs (4.9%), and thyroid supplements (4.7%). Overall, 38.3% of the participants reported receiving at least one medication regularly, with no significant difference (*Sig*. = 0.907) between inactivated virus (38.4%) and adenoviral vector (38%) group Table 1.

When asked about their COVID-19-related anamnesis, less than half of the participants (47.1%) reported being infected previously with no significant (*Sig*. = 0.534) difference between inactivated virus (48%) and adenoviral vector (45.6%) groups. Most of the infections occurred before vaccination (90.6%), while 9.4% after the second dose without a significant difference between the two vaccine platforms (*Sig*. = 0.625).

Less than half of the participants (48%) were inoculated against SARS-CoV-2 more than three months before the survey, while 33.7% were inoculated 1 to 3 months before the survey. Most of the participants (78.3%) received two doses, with no significant (*Sig*. = 0.138) difference between inactivated virus (80%) and adenoviral vector (75.22%) groups Table 2.

**Table 2:** COVID-19-related anamnesis of Algerian healthcare workers receiving COVID-19 vaccines (*n* = 721).

**Variable**	**Outcome**	**Inactivated virus** **vaccine (*n* = 450)**	**Adenoviral vector vaccine (*n* = 271)**	**Total** **(*n* = 721)**	***Sig***.
**Infection**	Yes	216 (48%)	125 (45.6%)	341 (47.1%)	0.534
	No	234 (52%)	149 (54.4%)	383 (52.9%)	
**Onset**	Before vaccination	197 (91.2%)	112 (89.6%)	309 (90.6%)	0.625
	After second dose	19 (8.8%)	13 (10.4%)	32 (9.4%)	
**Vaccination timing**	Less than a week ago	13 (2.9%)	12 (4.4%)	25 (3.5%)	0.274
	From a week to a month ago	64 (14.2%)	43 (15.9%)	107 (14.8%)	0.547
	From a month to 3 months ago	187 (41.6%)	56 (20.7%)	243 (33.7%)	**<** **0.001**
	More than 3 months ago	186 (41.3%)	160 (59%)	346 (48%)	**<** **0.001**
**Number of doses**	One dose ^‡^	90 (20%)	63 (24.8%)	153 (21.7%)	0.138
	Two doses	360 (80%)	191 (75.2%)	551 (78.3%)	

‡ *Chi-squared test (χ^2^) had been used with a significance level (Sig.) ≤ 0.05;, Participants who received Ad26.COV2.S were excluded*.

*Bold values refer to the statisitically signifcant values which are below 0.05*.

### Local Side Effects

**Figure 1:**
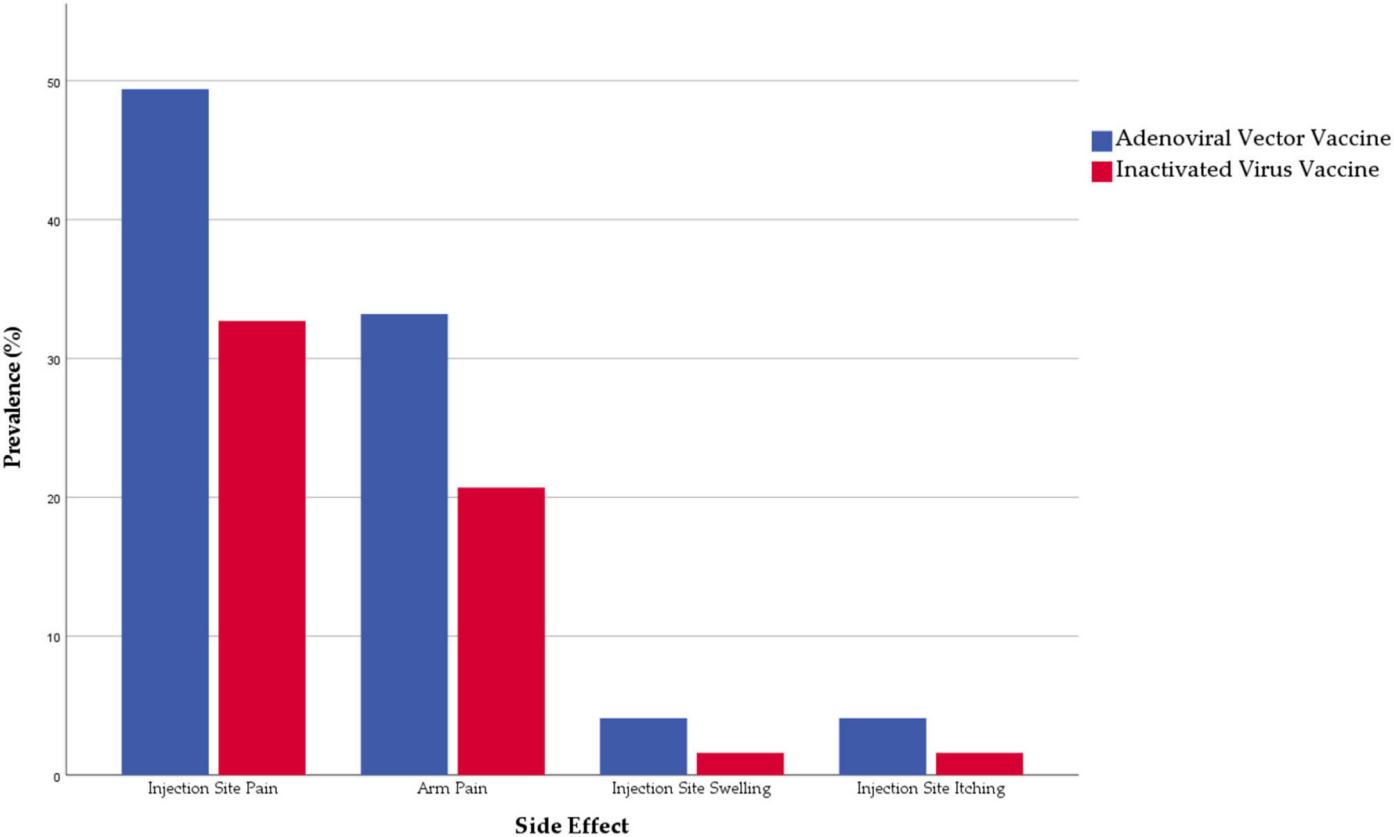
Prevalence of local side effects reported by Algerian healthcare workers (*n* = 721).

Less than half of the participants (49.1%) reported at least one local side effect (related to the injection site), with the adenoviral vector vaccines (61.3%) being more significantly (*Sig*. < 0.001) associated with local side effects than inactivated virus vaccine (41.8%). Injection site pain was the most common local side effect (39%), followed by arm pain (25.4%), and injection site swelling (2.5%) and itching (2.5%). Prevalence of all the solicited local side effects was significantly higher among the adenoviral vector group Figure 1.

Regarding their onset, most local side effects emerged 12 h (77.4%) with a significant (*Sig*. = 0.021) difference between inactivated virus (82.3%) and adenoviral vector (72%) vaccines. Local side effects needed a significantly shorter interval (earlier onset) among the inactivated virus group than the adenoviral virus group. Regarding their duration, most local side effects lasted for only 24 h (38.7%) or 24–72 h (46.7%), without significant differences between the inactivated virus and the adenoviral vector vaccines Table 3.

**Table 3:** Local side effects reported by Algerian healthcare workers receiving COVID-19 vaccines (*n* = 721).

**Variable**	**Outcome**	**Inactivated virus** **vaccine (*n* = 450)**	**Adenoviral vector vaccine (*n* = 271)**	**Total** **(*n* = 721)**	***Sig***.
**Local Side** **effects**	Injection site pain	147 (32.7%)	134 (49.4%)	281 (39%)	**<** **0.001**
	Arm pain	93 (20.7%)	90 (33.2%)	183 (25.4%)	**<** **0.001**
	Injection site swelling	7 (1.6%)	11 (4.1%)	18 (2.5%)	**0.037**
	Injection site itching	7 (1.6%)	11 (4.1%)	18 (2.5%)	**0.037**
	Total	188 (41.8%)	166 (61.3%)	354 (49.1%)	**<** **0.001**
**Onset**	≤ 12 h	153 (82.3%)	118 (72%)	271 (77.4%)	**0.021**
	> 12 h	33 (17.7%)	46 (28%)	79 (22.6%)	
**Duration**	24 h	77 (41.2%)	58 (35.8%)	135 (38.7%)	0.304
	From 24 to 72 h	82 (43.9%)	81 (50%)	163 (46.7%)	0.251
	From 3 days to a week	20 (10.7%)	16 (9.9%)	36 (10.3%)	0.802
	More than a week	8 (4.3%)	7 (4.3%)	15 (4.3%)	0.984

*Chi-squared test (χ^2^) had been used with a significance level (Sig.) ≤ 0.05*.

*Bold values refer to the statisitically signifcant values which are below 0.05*.

### Systemic Side Effects

**Figure 2:**
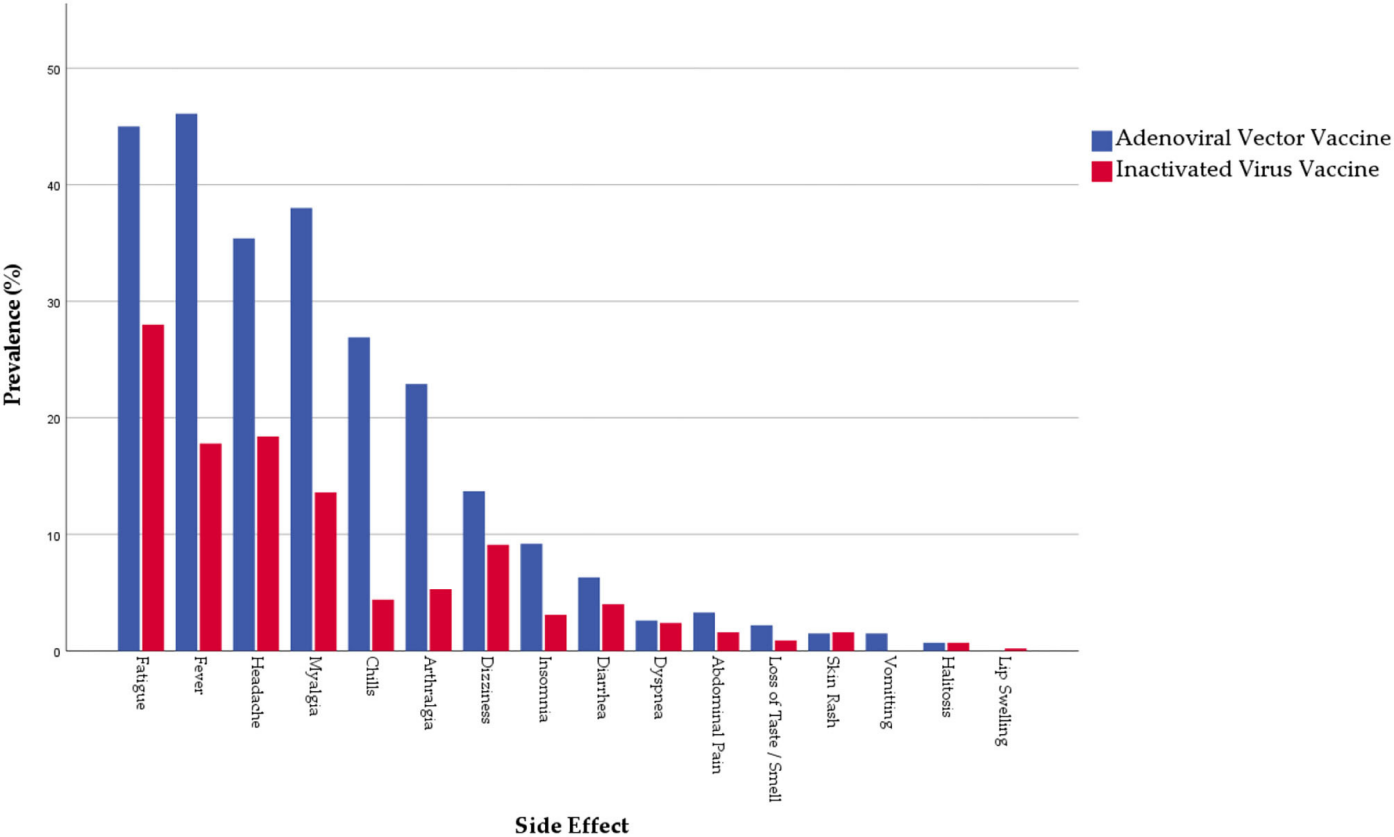
Prevalence of systemic side effects reported by Algerian healthcare workers (*n* = 721).

More than half of the participants (53.8%) reported at least one systemic side effect (not related to the injection site), with the adenoviral vector vaccines (68.3%) being more significantly (*Sig*. < 0.001) associated with systemic side effects than inactivated virus vaccine (45.1%). Fatigue was the most common systemic side effect (34.4%), followed by fever (28.4%), headache (24.8%), myalgia (22.7%), chills (12.9%), and arthralgia (11.9%). Prevalence of most solicited systemic side effects was significantly higher among the adenoviral vector group except for dizziness, diarrhea, dyspnoea, skin rash, and abdominal pain where the difference was not statistically significant despite being more frequent among the adenoviral vector group Figure 2.

Regarding their onset, most systemic side effects emerged within two weeks (81.5%), with a significant (*Sig*. < 0.001) difference between inactivated virus (73.6%) and adenoviral vector (89.7%) vaccines. Systemic side effects tended to require a shorter interval (earlier onset) to emerge among the inactivated virus group than the adenoviral virus group. Regarding their duration, most systemic side effects lasted for only 2 days (59.1%) or up to a week (28.5%), without significant differences between the inactivated virus and the adenoviral vector vaccines. Among all the participants, six reported seeking medical care after vaccination due to their side effects, five (1.1%) from the inactivated virus and one (0.4%) from the adenoviral vector group (*Sig*. = 0.418) Table 4.

**Table 4:** Systemic side effects reported by Algerian healthcare workers receiving COVID-19 vaccines (n = 721).

**Variable**	**Outcome**	**Inactivated virus** **vaccine (*n* = 450)**	**Adenoviral vector vaccine (*n* = 271)**	**Total** **(*n* = 721)**	***Sig***.
**Systemic Side** **effects**	Fever	80 (17.8%)	125 (46.1%)	205 (28.4%)	**<** **0.001**
	Headache	83 (18.4%)	96 (35.4%)	179 (24.8%)	**<** **0.001**
	Dizziness	41 (9.1%)	37 (13.7%)	78 (10.8%)	0.057
	Chills	20 (4.4%)	73 (26.9%)	93 (12.9%)	**<** **0.001**
	Fatigue	126 (28%)	122 (45%)	248 (34.4%)	**<** **0.001**
	Myalgia	61 (13.6%)	103 (38%)	164 (22.7%)	**<** **0.001**
	Arthralgia	24 (5.3%)	62 (22.9%)	86 (11.9%)	**<** **0.001**
	Diarrhea	18 (4%)	17 (6.3%)	35 (4.9%)	0.169
	Vomiting	0 (0%)	4 (1.5%)	4 (0.6%)	**0.020** ^*^
	Insomnia	14 (3.1%)	25 (9.2%)	39 (5.4%)	**<** **0.001**
	Dyspnea	11 (2.4%)	7 (2.6%)	18 (2.5%)	0.908
	Skin rash	7 (1.6%)	4 (1.5%)	11 (1.5%)	1.000 ^*^
	Loss of taste/smell	4 (0.9%)	6 (2.2%)	10 (1.4%)	0.189 ^*^
	Halitosis	3 (0.7%)	2 (0.7%)	5 (0.7%)	1.000 ^*^
	Lip swelling	1 (0.2%)	0 (0%)	1 (0.1%)	1.000 ^*^
	Abdominal pain	7 (1.6%)	9 (3.3%)	16 (2.2%)	0.119
	Total	203 (45.1%)	185 (68.3%)	388 (53.8%)	**<** **0.001**
**Onset**	Immediately	28 (14.5%)	16 (8.6%)	44 (11.6%)	**<** **0.001**
	Within 2 week	142 (73.6%)	166 (89.7%)	308 (81.5%)	**<** **0.001**
	After 2 weeks	23 (11.9%)	3 (1.6%)	26 (6.9%)	**<** **0.001**
**Duration**	2 days	106 (55.5%)	112 (62.9%)	218 (59.1%)	0.147
	From 2 days to a week	54 (28.3%)	51 (28.7%)	105 (28.5%)	0.936
	From a week to 2 weeks	12 (6.3%)	7 (3.9%)	19 (5.1%)	0.307
	From 2 weeks to 4 weeks	8 (4.2%)	3 (1.7%)	11 (3%)	0.158
	More than 4 weeks	11 (5.8%)	5 (2.8%)	16 (4.3%)	0.164
**Medical care**	Yes	5 (1.1%)	1 (0.4%)	6 (0.8%)	0.418 ^*^
	No	445 (98.9%)	270 (99.6%)	715 (99.2%)	

(*) *Chi-squared test (χ^2^) and Fisher's-exact test; had been used with a significance level (Sig.) ≤ 0.05*.

*Bold values refer to the statisitically signifcant values which are below 0.05*.

When asked about how they managed their post-vaccination side effects, 38.1% of the participants reported taking medications to manage their side effects. The adenoviral vector group (52%) was significantly (*Sig*. < 0.001) more associated with post-vaccination medications than the inactivated virus (29.8%) group. The most used medication was Paracetamol (36.9%) and to a lesser extent Aspirin (2.1%). (Table 5).

**Table 5:** Post-vaccination medications received by Algerian healthcare workers (*n* = 721).

**Variable**	**Inactivated virus vaccine (*n* = 450)**	**Adenoviral vector** **vaccine (*n* = 271)**	**Total (*n* = 721)**	***Sig***.
**Paracetamol**	129 (28.7%)	137 (50.6%)	266 (36.9%)	**<** **0.001**
**Aspirin**	6 (1.3%)	9 (3.3%)	15 (2.1%)	0.070
**Total**	134 (29.8%)	141 (52%)	275 (38.1%)	**<** **0.001**

*Chi-squared test (χ^2^) had been used with a significance level (Sig.) ≤ 0.05*.

*Bold values refer to the statisitically signifcant values which are below 0.05*.

### Risk Factors of Post-vaccination Side Effects

Females had significantly higher levels of overall side effects (71.6 vs. 55.3%), local side effects (58.5 vs. 35.6%), and systemic side effects (59.4 vs. 45.8%) than males, respectively. The local side effects were the most common among the age group of 31–40 years-old (56.8%), followed by the age group of 41–50 years-old (52.4%); on the other hand, the systemic side effects were the most common the age group of over 60 years-old (75%), followed by the age group of 51–60 years-old (66.7%).

Prevalence of local (51.3 vs. 32.6%) and systemic (55.1 vs. 44.2%) side effects was higher among non-smokers than smokers; while allergic participants had significantly higher prevalence of local (63.3 vs. 42.9%) and systemic (67 vs. 48.1%) side effects than their counterparts, respectively. The participants who reported suffering from at least one chronic illness had a significantly higher prevalence of local (57.7 vs. 46.5%) and systemic (61.3 vs. 51.5%) side effects than their counterparts, respectively. Similarly, the participants who reported taking medications regularly had a significantly higher prevalence of local (59.4% vs. 42.7%) and systemic (62 vs. 48.8%) side effects than their counterparts, respectively.

Previous COVID-19 infection was significantly associated with a higher prevalence of local (53.5 vs. 44.1%) and systemic (59.5 vs. 47.3%) side effects. Similarly, receiving two doses was significantly associated with a higher prevalence of local (52.1 vs. 39.4%) and systemic (56.4 vs. 45.3%) side effects compared with receiving one dose, respectively Table 6.

**Table 6:** Risk factors of post-vaccination side effects reported by Algerian healthcare workers (*n* = 721).

**Variable**	**Outcome**	**Local SE**	***Sig***.	**Systemic SE**	***Sig***.	**Total SE**	***Sig***.
**Sex**	Female	249 (58.5%)	**<** **0.001**	253 (59.4%)	**<** **0.001**	305 (71.6%)	**<** **0.001**
	Male	105 (35.6%)		135 (45.8%)		163 (55.3%)	
**Age**	20–30 years-old	121 (42.6%)	**0.005**	112 (39.4%)	**<** **0.001**	151 (53.2%)	**<** **0.001**
**group**	31–40 years-old	130 (56.8%)	**0.005**	145 (63.3%)	**<** **0.001**	169 (73.8%)	**<** **0.001**
	41–50 years-old	66 (52.4%)	0.417	75 (59.5%)	0.157	87 (69%)	0.284
	51–60 years-old	29 (43.9%)	0.379	44 (66.7%)	**0.028**	48 (72.7%)	0.163
	> 60 years-old	8 (50%)	0.942	12 (75%)	0.086	13 (81.3%)	0.166
**Tobacco**	Smoker	28 (32.6%)	**0.001**	38 (44.2%)	0.056	47 (54.7%)	**0.034**
**smoking**	Non-smoker	326 (51.3%)		350 (55.1%)		421 (66.3%)	
**Allergy**	Yes	138 (63.3%)	**<** **0.001**	146 (67%)	**<** **0.001**	175 (80.3%)	**<** **0.001**
	No	216 (42.9%)		242 (48.1%)		293 (58.3%)	
**Chronic**	Yes	97 (57.7%)	**0.011**	103 (61.3%)	**0.026**	120 (71.4%)	**0.043**
**illnesses**	No	257 (46.5%)		285 (51.5%)		348 (62.9%)	
**Medications**	Yes	164 (59.4%)	**<** **0.001**	171 (62%)	**<** **0.001**	204 (73.9%)	**<** **0.001**
	No	190 (42.7%)		217 (48.8%)		264 (59.3%)	
**Infection**	Yes	205 (53.5%)	**0.011**	228 (59.5%)	**0.001**	270 (70.5%)	**<** **0.001**
	No	149 (44.1%)		160 (47.3%)		198 (58.6%)	
**Number of doses**	One dose	67 (39.4%)	**0.004**	77 (45.3%)	**0.011**	92 (54.1%)	**<** **0.001**
	Two doses	287 (52.1%)		311 (56.4%)		376 (68.2%)	

*Chi-squared test (χ^2^) had been used with a significance level (Sig.) ≤ 0.05*.

*Bold values in all tables refer to the statisitically signifcant values which are below 0.05*.

The participants who suffered from allergy (80.3 vs. 58.3%) and chronic obstructive pulmonary disease (87.9 vs. 63.8%) had significantly higher prevalence of post-vaccination side effects compared with their counterparts who did not report these diseases. Similarly, the participants who reported taking anti-asthmatic (81.6 vs. 64%), antihistaminic (76.8% *vs*. 62.7%), anti-reflux (83.3 vs. 63.9%), and thyroid hormone (82.4 vs. 64%) had significantly higher prevalence of post-vaccination side effects compared with their counterparts who did not report using these medications regularly.

### Regression Analysis

Multivariate logistic regression was performed to analyse the demographic and anamnestic risk factors of post-vaccination side effects. For the inactivated virus vaccine, being a female (adjusted odds ratio “AOR”: 2.500; confidence interval “CI” 95%: 1.579–3.959), suffering from allergy (AOR: 3.487; CI 95%: 2.061–5.901) and being infected previously with COVID-19 (AOR: 2.373; CI 95%: 1.555–3.621) had significantly higher odds of experiencing post-vaccination side effects in general. Compared to the youngest age group (20–30 years-old), all age groups had higher odds for experiencing side effects. Smoking and being disease-free were associated with lower odds but without statistical significance Table 7.

**Table 7:** Logistic regression of risk factors for inactivated virus vaccine side effects reported by Algerian healthcare workers (*n* = 450).

**Predictor**	**B (SE)**	**Wald**	**AOR**	**CI 95%**	***Sig***.
Sex: female (vs. male)	0.916 (0.234)	15.288	2.500	1.579–3.959	**<** **0.001**
Age group: 31–40 yo (vs. 20–30 yo)	0.601 (0.255)	5.552	1.823	1.106–3.004	**0.018**
Age group: 41–50 yo (vs. 20–30 yo)	0.231 (0.312)	0.551	1.260	0.684–2.323	0.458
Age group: 51–60 yo (vs. 20–30 yo)	– 0.028 (0.430)	0.004	0.972	0.418–2.261	0.948
Age group: > 60 yo (vs. 20–30 yo)	0.636 (0.930)	0.468	1.890	0.305–11.692	0.494
Tobacco: smoker (vs. non-smoker)	– 0.067 (0.356)	0.036	0.935	0.466–1.877	0.850
Allergy: yes (vs. no)	1.249 (0.268)	21.666	3.487	2.061–5.901	**<** **0.001**
Non-communicable disease: yes (vs. no)	0.125 (0.309)	0.163	1.133	0.618–2.078	0.686
Medications: yes (vs. no)	– 0.158 (0.280)	0.316	0.854	0.493–1.480	0.574
Previous infection: yes (vs. no)	0.864 (0.216)	16.067	2.373	1.555–3.621	**<** **0.001**
Number of doses: two (vs. one)	0.312 (0.260)	1.431	1.366	0.820–2.275	0.232

*Bold values refer to the statisitically signifcant values which are below 0.05*.

For the adenoviral vector vaccines, being a female (AOR: 2.503; CI 95%: 1.216–5.512) had significantly higher odds of experiencing post-vaccination side effects in general. Compared to the youngest age group (20–30 years-old), all other age groups had higher odds for experiencing side effects. Smoking and being disease-free were associated with lower odds but without statistical significance Table 8.

**Table 8:** Logistic regression of risk factors for adenoviral vector vaccine side effects reported by Algerian healthcare workers (*n* = 271).

**Predictor**	**B (SE)**	**Wald**	**AOR**	**CI 95%**	***Sig***.
Sex: female (vs. male)	0.917 (0.368)	6.200	2.503	1.216–5.512	**0.013**
Age group: 31–40 yo (vs. 20–30 yo)	1.576 (0.409)	14.839	4.837	2.169–10.785	**<** **0.001**
Age group: 41–50 yo (vs. 20–30 yo)	1.140 (0.495)	5.300	3.127	1.185–8.254	**0.021**
Age group: 51–60 yo (vs. 20–30 yo)	2.086 (0.825)	6.393	8.054	1.598–40.580	**0.011**
Age group: > 60 yo (vs. 20–30 yo)	1.582 (1.204)	1.727	4.863	0.460–51.443	0.189
Tobacco: smoker (vs. non-smoker)	0.199 (0.501)	0.158	1.220	0.457–3.258	0.691
Allergy: yes (vs. no)	0.122 (0.438)	0.078	1.130	0.479–2.665	0.780
Non-communicable disease: yes (vs. no)	– 0.473 (0.555)	0.727	0.623	0.210–1.849	0.394
Medications: yes (vs. no)	0.606 (0.461)	1.728	1.834	0.743–4.528	0.189
Previous infection: yes (vs. no)	0.508 (0.354)	2.058	1.662	0.830–3.327	0.151
Number of Doses: two (vs. one)	0.642 (0.358)	3.213	1.901	0.942–3.836	0.073

*Bold values refer to the statisitically signifcant values which are below 0.05*.

## Discussion

In the present work, an online survey-based study was carried out to evaluate the post-vaccination side effects among healthcare workers who received COVID-19 vaccines and their related risk factors in Algeria. The reported side effects were compared between inactivated (BBIBP-CorV and CoronaVac) and adenoviral vector-based (AZD1222, Gam-COVID-Vac and Ad26.COV2.S) vaccines approved in Algeria. In fact, healthcare workers were among the prioritized groups for COVID-19 vaccine in Algeria. Also, their professional background guaranteed a better and more detailed description of the post-vaccination side effects. For these reasons, multiple studies were conducted to determine vaccines side effects among this population subset in different countries, e.g., Czech Republic, Germany, Jordan, Saudi Arabia, Slovakia, Turkey, and United Arab Emirates (19–28).

Overall, 49.1, and 53.8% of the surveyed healthcare workers in our study reported at least one local or systemic side effect, respectively. The local and systemic side effects were significantly more frequent among the adenoviral vector vaccines group (61.3, and 68.3%) than the inactivated virus vaccinated group (41.8, and 45.1%). This finding is consistent with the results of multiple previous studies that reported that the Chinese inactivated vaccines, i.e., BBIBP-CorV and CoronaVac induced fewer side effects than either adenoviral vector-based mRNA-based vaccines (18, 19, 40–42). Moreover, the reported side effects were generally mild in patients who received inactivated vaccines (19, 22, 41–44). The side effects duration was longer in BBIBP-CorV than in the mRNA-based vaccines (43). The local and systemic side effects were more prevalent after the second dose than the first dose for both inactivated and adenoviral vector vaccines, thus, confirming what was previously reported in different studies (45, 46). Contrarily, Omeish et al. 2021 in Jordan and Jeon et al. 2021 in Korea found that side effects were more frequent and more severe after the first dose (18, 47).

The most common local side effects in this study was injection site pain (39%), followed by arm pain (25.4%), and injection site swelling (2.5%) and itching (2.5%). However, these side effects emerged generally with low frequencies than previously reported, especially with the adenoviral vector vaccines, i.e., AZD1222 where injection site pain was reported with a prevalence higher than 58% (24, 27, 47–49). Similarly, a large-scale multinational study covering more than 10,000 vaccinees in the Arab countries reported that more than 58% of the participants suffered from injection site pain and swelling (50).

In our study, the local side effects generally appeared earlier among the inactivated virus group than the adenoviral vector group, and they generally resolved within the first day (38.7%) or between the first and third day (46.7%) post-vaccination in the two groups. This finding is in consistence with what Solomon et al. 2021 reported, where most of the AZD1222 recipients developed injection site pain within the first 12 h post-vaccination and disappeared between the first and the third day (49).

Regarding systemic side effects, the most commonly reported ones were fatigue (34.4%), fever (28.4%), headache (24.8%) and myalgia (22.7%). These symptoms with chills and dizziness are the most common reported side effects for all available vaccines and are generally reported with higher frequency than in our study, especially for adenoviral vector vaccines. (19, 21, 24). For instance, fatigue, fever and headache were reported by 90%, 66% and 62% of vaccinated individuals in Saudi Arabia following AZD1222 (21). In the same Saudi study, it was also reported that 75% of the systemic adverse effects lasted for 1 day (21). In our study, the systemic side effects generally emerged in the first day and lasted mostly for 2 days.

Additionally, 38.1% of our participants took post-vaccination medications, mainly Paracetamol, to manage these side effects and 1.1% reported being hospitalized, thus, confirming the mildness of these side effects. In Iraq, 57.2% of the vaccinated healthcare workers took Paracetamol, especially among those vaccinated with BNT162b2 and AZD1222, and 8.7% of them sought medical care (42).

The second objective of this study was to determine the risk factors related to the emergence of post-vaccination side effects. Our results showed that sex, age, tobacco, allergy, chronic diseases, regular medications and previous infection with COVID-19 were associated with the frequency of these side effects.

Being a female increased significantly the risk of developing side effects for both inactivated virus vaccines (OR = 2.641; CI 95% = 1.780–3.919) and adenoviral vector vaccines (OR = 2.002; CI 95% = 1.113–3.601). The same observation was also reported not only for COVID-19 vaccines but also for other bacterial and viral vaccines in which females were more likely to develop side effects signs than males (19, 28, 51–53). These results are unsurprising because of the hormonal and genetic differences between males and females, leading to different immunological reactions (54). Di Resta et al. 2021 reported that the antibody titer in BNT162b2 recipients was higher in female healthcare workers, which was associated with high side effects frequency (55).

Regarding age, our results showed that the young healthcare workers (20–30 years-old) had developed less frequent local and systemic side effects than the older ones for the two vaccine groups. Moreover, the most exposed to these side effects was the category of 30–50 years old. Our results are generally in line with multiple previous studies despite some differences in age categorization. Menni et al. 2021 reported a high frequency of post-vaccination side effects following mRNA-based and adenoviral vector-based vaccines the people under 55 years old (56). Similarly, other studies found the same observation for a younger individual of <49 years (Czech Republic), (23) <45 years (Jordan), (20) <39 years (Germany), (57) <38 years (Iran), (51) and <32 years (Turkey) (28) for both inactivated virus and adenoviral vector vaccines. In addition, Klugar et al. 2021 reported that the post-vaccination side effects were more reported in younger healthcare workers who received mRNA-based vaccines, i.e., BNT162b2 and adenoviral vector vaccine, i.e. AZD1222 (24).

Our participants with chronic diseases did not develop more side effects than those without chronic diseases for the two vaccinated groups. Contrarily, allergic individuals and those taking medications regularly developed significantly more side effects than their counterparts. This result supports the observation reported by Alhazmi et al. 2021 in Saudi Arabia, while other studies found that persons with chronic conditions and regular medication are more likely to develop side effects (21, 23, 24, 27, 28). For the association between regular medications and side effects, it is imperative to deal with this finding cautiously since the reported medications are various and include antihistaminic agents, anti-diabetics, antihypertensive drugs, contraceptives, and thyroid hormones and little is known about their interaction with the different COVID-19 vaccines. In the previous studies that found a lower prevalence of side effects among people with chronic diseases, this finding was attributed to their weak immune system, which leads to a weaker immune response (46).

The history of infection with COVID-19 increased significantly the risk of developing side effects even in both vaccine groups. The same results were found in multiple previous studies for different COVID-19 vaccines, including the mRNA-based ones (23, 24, 27, 28, 50, 57). Moreover, the antibody titer after COVD-19 vaccination was higher among individuals with a past history of SARS-CoV-2 infection than those who had not been in contact with this pathogen (44). On the contrary, two Saudi Arabia studies failed to find any association between the history of COVID-19 infection and post-vaccination side effects prevalence and severity (21, 46). Nevertheless, Zare et al. 2021 found a significant association between previous infection and post-vaccination side effects prevalence in the group of Gam-COVID-Vac but not in the group of AZD1222 (58). This finding should be however interpreted cautiously since the period between the COVID-19 recovery and the date of vaccination is unknown.

### Limitations

At last, this study has several limitations related to the sample selection and the survey method. The survey was conducted using convenient and snowball sampling based on an online questionnaire that could marginalize individuals without access to the internet and overrepresent younger individuals who tend to spend more time with social media. Given the increase in familywise error rate across the reported statistical analyses, lack of control can be considered one of the limitations of this study findings. Another limitation is the lower number of healthcare workers who received vector-based vaccines; this could be explained by the fact that the inactivated vaccines are the most used and the most preferred vaccines by the Algerian population, as described in previous studies.

### Strengths

To the best of the authors' knowledge, this study provides the first evidence about self-reported COVID-19 vaccines side effects among the Algerian population. It also provides a cross-vaccine comparison for the inactivated virus versus adenoviral vector vaccines.

## Conclusion

In conclusion, this is the first study that concerns COVID-19 vaccines among healthcare workers in Algeria. Results showed that local and systemic are generally more prevalent with adenoviral vector vaccines than inactivated virus vaccines. Injection site pain (39%) and arm pain (25.4%) were the most common local side effects, while fatigue (34.4%), fever (28.4%), headache (24.8%) and myalgia (22.7%) were the most reported systemic side effects. Females, allergic individuals, and those with a history of COVID-19 infection had a significantly higher risk of developing post-vaccination side effects for either inactivated virus or adenoviral vector vaccines.

## Data Availability Statement

The data that support the findings of this study are available from the corresponding author upon reasonable request.

## Ethics Statement

The studies involving human participants were reviewed and approved by the study was conducted according to the guidelines of the Declaration of Helsinki and approved by the Ethics Committee of the Faculty of Natural and Life Sciences, University of Djelfa on 20 October 2021 with reference number 117/10/2021. The patients/participants provided their written informed consent to participate in this study.

## Author Contributions

ML: conceptualization, validation, and project administration. ML and AR: methodology and writing—original draft preparation. AR: formal analysis and supervision. ML, MR, DB, HA, and AO: investigation. JK and MK: writing—review and editing. AP and MK: funding acquisition. All authors contributed to the article and approved the submitted version.

## Funding

This study was supported by Masaryk University grants no. MUNI/IGA/1104/2021 and MUNI/A/1402/2021. The INTER-EXCELLENCE grant number LTC20031 supported the work of AR, JK, AP, and MK — Toward an International Network for Evidence-based Research in Clinical Health Research in the Czech Republic.

## Conflict of Interest

The authors declare that the research was conducted in the absence of any commercial or financial relationships that could be construed as a potential conflict of interest.

## Publisher's Note

All claims expressed in this article are solely those of the authors and do not necessarily represent those of their affiliated organizations, or those of the publisher, the editors and the reviewers. Any product that may be evaluated in this article, or claim that may be made by its manufacturer, is not guaranteed or endorsed by the publisher.
