# Supplementary material for: Side Effects of COVID-19 Inactivated Virus vs. Adenoviral Vector Vaccines: Experience of Algerian Healthcare Workers
Source: Front Public Health. 2022 May 16;10:896343. doi: 10.3389/fpubh.2022.896343 (PMC9149155; doi:10.3389/fpubh.2022.896343)
Supplement: Supplementary file 1 [file Table_1.DOCX]

**Table S1.** Sociodemographic Characteristics of Algerian Healthcare Workers Receiving COVID-19 Vaccines (*n* = 721).

| **Variable** | **Outcome** | **Inactivated Virus**  **Vaccine (*n* = 450)** | **Adenoviral Vector**  **Vaccine (*n* = 271)** | **Total**  **(*n* = 721)** | ***Sig*.** |
| --- | --- | --- | --- | --- | --- |
| **Sex** | Female | 286 (63.6%) | 140 (51.7%) | 426 (59.1%) | **0.002** |
|  | Male | 164 (36.4%) | 131 (48.3%) | 295 (40.9%) |  |
| **Age Group** | 20 – 30 years-old | 199 (44.2%) | 85 (31.4%) | 284 (39.4%) | **< 0.001** |
|  | 31 – 40 years-old | 130 (28.9%) | 99 (36.5%) | 229 (31.8%) | **0.033** |
|  | 41 – 50 years-old | 78 (17.3%) | 48 (17.7%) | 126 (17.5%) | 0.897 |
|  | 51 – 60 years-old | 36 (8%) | 30 (11.1%) | 66 (9.2%) | 0.166 |
|  | > 60 years-old | 7 (1.6%) | 9 (3.3%) | 16 (2.2%) | 0.119 |
| **Marital Status** | Single | 213 (48.1%) | 108 (40.3%) | 321 (45.1%) | **0.043** |
|  | Married | 229 (51.7%) | 158 (59%) | 387 (54.4%) | 0.060 |
|  | Divorced/Widow | 1 (0.2%) | 2 (0.7%) | 3 (0.4%) | 0.560 * |
| **Profession** | Physician | 134 (29.8%) | 122 (45%) | 256 (35.5%) | **< 0.001** |
|  | Dentist | 101 (22.4%) | 46 (17%) | 147 (20.4%) | 0.077 |
|  | Pharmacist | 35 (7.8%) | 18 (6.6%) | 53 (7.4%) | 0.571 |
|  | Nurse | 41 (9.1%) | 26 (9.6%) | 67 (9.3%) | 0.829 |
|  | Paramedic | 45 (10%) | 22 (8.1%) | 67 (9.3%) | 0.399 |
|  | Lab Worker | 20 (4.4%) | 8 (3%) | 28 (3.9%) | 0.315 |
|  | Midwife | 14 (3.1%) | 3 (1.1%) | 17 (2.4%) | 0.086 |
|  | Psychologist | 3 (0.7%) | 2 (0.7%) | 5 (0.7%) | 1.000 * |
|  | Physiotherapist | 0 (0%) | 1 (0.4%) | 1 (0.1%) | 0.376 * |
|  | Administrative Staff | 19 (4.2%) | 13 (4.8%) | 32 (4.4%) | 0.717 |
|  | Student | 38 (8.4%) | 10 (3.7%) | 48 (6.7%) | **0.013** |
| **Sector** | Public | 343 (76.2%) | 214 (79%) | 557 (77.3%) | 0.394 |
|  | Private | 107 (23.8%) | 57 (21%) | 164 (22.7%) |  |
| **City** | Alger | 120 (26.7%) | 62 (22.9%) | 184 (25.2%) | 0.257 |
|  | Blida | 27 (6.0%) | 14 (5.2%) | 41 (5.7%) | 0.640 |
|  | Tebessa | 18 (4.0%) | 17 (6.3%) | 35 (4.8%) | 0.169 |
|  | Oran | 25 (5.6%) | 9 (3.3%) | 34 (4.7%) | 0.170 |
|  | Sétif | 18 (4.0%) | 11 (4.1%) | 29 (4.0%) | 0.969 |
|  | Annaba | 15 (3.3%) | 13 (4.8%) | 28 (3.9%) | 0.324 |
|  | Constantine | 19 (4.2%) | 7 (2.6%) | 26 (3.6%) | 0.253 |
|  | Batna | 13 (2.9%) | 11 (4.1%) | 24 (3.3%) | 0.396 |
|  | Tizi Ouzou | 15 (3.3%) | 6 (2.2%) | 21 (2.9%) | 0.387 |
|  | Béjaia | 16 (3.6%) | 5 (1.8%) | 21 (2.9%) | 0.186 |
|  | Tlemcen | 10 (2.2%) | 11 (4.4%) | 21 (2.9%) | 0.155 |
|  | Bouira | 9 (2.0%) | 7 (2.6%) | 16 (2.2%) | 0.607 |
|  | Sidi Belabbas | 9 (2.0%) | 6 (2.2%) | 15 (2.1%) | 0.845 |
|  | Ain Defla | 11 (2.4%) | 4 (1.5%) | 15 (2.1%) | 0.378 |
|  | Tipaza | 8 (1.8%) | 6 (2.2%) | 14 (1.9%) | 0.681 |
|  | Djelfa | 5 (1.1%) | 8 (3.0%) | 13 (1.8%) | 0.086 * |
|  | Médéa | 6 (1.3%) | 5 (1.8%) | 11 (1.5%) | 0.755 * |
|  | Biskra | 6 (1.3%) | 3 (1.1%) | 9 (1.2%) | 1.000 * |
|  | Jijel | 8 (1.8%) | 1 (0.4%) | 9 (1.2%) | 0.164 * |
|  | Chlef | 6 (1.3%) | 2 (0.7%) | 8 (1.1%) | 0.717 * |
|  | Bordj Bou Arreridj | 6 (1.3%) | 2 (0.7%) | 8 (1.1%) | 0.717 * |
|  | Mascara | 6 (1.3%) | 2 (0.7%) | 8 (1.1%) | 0.717 * |
|  | Laghouat | 3 (0.7%) | 5 (1.8%) | 8 (1.1%) | 0.160 * |
|  | Oum El Bouaghi | 3 (0.7%) | 5 (1.8%) | 8 (1.1%) | 0.160 * |
|  | Mila | 6 (1.3%) | 2 (0.7%) | 8 (1.1%) | 0.717 * |
|  | Guelma | 5 (1.1%) | 3 (1.1%) | 8 (1.1%) | 1.000 * |
|  | Tiaret | 5 (1.1%) | 3 (1.1%) | 8 (1.1%) | 1.000 * |
|  | Skikda | 3 (0.7%) | 4 (1.5%) | 7 (1.0%) | 0.435 * |
|  | Msila | 6 (1.3%) | 1 (0.4%) | 7 (1.0%) | 0.265 * |
|  | Saida | 2 (0.4%) | 4 (1.5%) | 6 (0.8%) | 0.205 * |
|  | Boumerdes | 3 (0.7%) | 3 (1.1%) | 6 (0.8%) | 0.677 * |
|  | Souk Ahras | 2 (0.4%) | 4 (1.5%) | 6 (0.8%) | 0.205 * |
|  | Ain Temouchent | 3 (0.7%) | 3 (1.1%) | 6 (0.8%) | 0.677 * |
|  | Tamenasset | 3 (0.7%) | 2 (0.7%) | 5 (0.7%) | 1.000 * |
|  | Nâama | 3 (0.7%) | 2 (0.7%) | 5 (0.7%) | 1.000 * |
|  | Mostaghanem | 3 (0.7%) | 2 (0.7%) | 5 (0.7%) | 1.000 * |
|  | Béchar | 2 (0.4%) | 2 (0.7%) | 4 (0.6%) | 0.634 * |
|  | Tindouf | 3 (0.7%) | 1 (0.4%) | 4 (0.6%) | 1.000 * |
|  | Ghelizane | 2 (0.4%) | 2 (0.7%) | 4 (0.6%) | 0.634 * |
|  | El Oued | 2 (0.4%) | 2 (0.7%) | 4 (0.6%) | 0.634 * |
|  | Al Taref | 2 (0.4%) | 1 (0.4%) | 3 (0.4%) | 1.000 * |
|  | Tissemsilt | 3 (0.7%) | 0 (0%) | 3 (0.4%) | 0.295 * |
|  | Adrar | 1 (0.2%) | 2 (0.7%) | 3 (0.4%) | 0.560 * |
|  | Khenchela | 1 (0.2%) | 2 (0.7%) | 3 (0.4%) | 0.560 * |
|  | Ghardaya | 2 (0.4%) | 1 (0.4%) | 3 (0.4%) | 1.000 * |
|  | Touggourt | 1 (0.2%) | 1 (0.4%) | 2 (0.3%) | 1.000 * |
|  | Ouargla | 1 (0.2%) | 1 (0.4%) | 2 (0.3%) | 1.000 * |
|  | In Guezzam | 1 (0.2%) | 0 (0%) | 1 (0.1%) | 1.000 * |
|  | El Meghaier | 1 (0.2%) | 0 (0%) | 1 (0.1%) | 1.000 * |
|  | Timimoune | 1 (0.2%) | 0 (0%) | 1 (0.1%) | 1.000 * |
|  | El Beyedh | 0 (0%) | 1 (0.4%) | 1 (0.1%) | 0.376 * |
|  | Illizi | 1 (0.2%) | 0 (0%) | 1 (0.1%) | 1.000 * |

Chi-squared test (*χ^2^*) and Fisher’s-exact test (*) had been used with a significance level (*Sig*.) ≤ 0.05.
